# Strategic Decision-Making Learning from Label Distributions: An Approach for Facial Age Estimation

**DOI:** 10.3390/s16070994

**Published:** 2016-06-28

**Authors:** Wei Zhao, Han Wang

**Affiliations:** School of Electrical and Electronic Engineering, Nanyang Technological University, 50 Nanyang Avenue, Singapore 639798; zhao0183@e.ntu.edu.sg

**Keywords:** strategic decision-making, label distribution learning, facial image, age estimation

## Abstract

Nowadays, label distribution learning is among the state-of-the-art methodologies in facial age estimation. It takes the age of each facial image instance as a label distribution with a series of age labels rather than the single chronological age label that is commonly used. However, this methodology is deficient in its simple decision-making criterion: the final predicted age is only selected at the one with maximum description degree. In many cases, different age labels may have very similar description degrees. Consequently, blindly deciding the estimated age by virtue of the highest description degree would miss or neglect other valuable age labels that may contribute a lot to the final predicted age. In this paper, we propose a strategic decision-making label distribution learning algorithm (SDM-LDL) with a series of strategies specialized for different types of age label distribution. Experimental results from the most popular aging face database, FG-NET, show the superiority and validity of all the proposed strategic decision-making learning algorithms over the existing label distribution learning and other single-label learning algorithms for facial age estimation. The inner properties of SDM-LDL are further explored with more advantages.

## 1. Introduction

Recently, with the expanding popularity of Microsoft’s “How-old.net” [1] all over the world and also the rapid development of computer vision, pattern recognition and biometrics, more and more attention has been paid to human facial age estimation, which is utilized in the scenarios where an individual’s age needs to be obtained without specifically identifying other irrelevant personal information, such as electronic customer relationship management [2,3], human–computer interaction (HCI) [4], security surveillance monitoring [5,6], age-based visual advertisement and even entertainment.

Unlike other face-oriented problems, the difficulties of computer-based facial age estimation [7,8] are reflected in the following aspects:

1. Difference of aging process: Different people have their own living environment, ethnic group, gender, lifestyle, social contact, health condition and even gene diversity, which all together determine the speed of aging.
2. Shape or texture: Different forms of aging will emerge at different age levels. For example, from infancy to adolescence, the craniofacial growth (shape growth) is the main change. However, from adult period to old age, the craniofacial change decreases remarkably and skin transformation (texture change) would be the most prominent change.
3. Data insufficiency: It takes great effort to search and collect old photos which were taken years ago. As a result, it is rather difficult for almost everyone to find one photo in each past year, let alone requiring the same shooting angle, lighting condition, resolution and background. In addition, only the past and present photos might be available, which means it is quite infrequent that a complete set of a person’s facial images with each age label can be gathered before his or her life ends. On the other hand, aging is a process which takes place moment by moment, so it is impossible to obtain multiple facial images for one person at the same time of different years. In fact, we only have a very limited number of aging datasets, especially that can cover the entire age range and are evenly distributed.
4. Disturbance: Some females tend to show their younger faces, so final estimation results will be largely interfered with by using cosmetics and accessories.

A lot of facial age estimation approaches have been put forward, some of which are able to obtain rather satisfying performance. Among them, most of the traditional approaches formulate facial age estimation problem based on classification [9,10,11,12], regression [5,13,14,15] or a combination of the two. Suppose we have a dataset of N training samples, $\left\{\left(x_{i},y_{i}\right)|i=1,...,N\right\}$, in which $x_{i}$ represents the ith face image and $y_{i}$ represents the corresponding age label. In multi-class classification, every sample will be regarded as a single independent age label for training; as a result, we get a multi-classifier to estimate a person’s age. However, the problem is, the age labels have no relationship with each other, i.e., each age label is only treated as a separate entity in the training process while, in essence, human age labels are sequential. Thus, this kind of multi-classification method may omit some connotative information of the correlation among different age labels, which together compose the fine-ordered age set. For instance, two images with adjacent age labels for the same person will be more similar than those with far-apart labels. In short, multi-class classification cannot take full advantage of the correlation among ordinal age labels. In contrast, the regression method aims to find the best mapping from raw images to the corresponding ages and get a function for age estimation. However, craniofacial and skin changes at different age levels would result in an unstable random process in feature space, so the kernels used to assess the similarities among different ages could drift. As for the estimation performance, it has been shown in the literature [5,14,16] that when different datasets are used for training and testing, the regression method will show better or worse results than the classification-based method. In addition, Guo et al. [5,17] proposed a hybrid method that combines classification and regression approaches together to make use of both advantages. As a result, the actual performance is further improved to some degree. However, it is well known that the aging process is diversified for different age levels. As an analogy, the aging process from 22 to 25 would have a different tendency compared to that from 62 to 65. Therefore, it is more credible to compare two age labels’ relative sequence (smaller or larger) than the differences among labels. Inspired by the aforementioned defects, ordinal hyperplanes ranker (OHRank) [18] based age estimation was proposed based on an ordinal hyperplane ranking algorithm that splits the estimation task into several cost-sensitive binary classification subproblems.

In spite of all the above merits, these algorithms fail to consider that age labels have a certain relationship with each other to different degrees. More specifically, because aging is a slow and gradually varied process, the adjacent labels around a certain age label X will necessarily have a connection with label X and thus can inevitably describe the characteristics of label X to different degrees. Thus, in [19], Yan et al. proposed a solution to the age ranking problem based on the training samples with uncertain age labels: a small range (within one year) was set as the uncertain labels for a given age label. In [20], the *label sensitive* concept was proposed for better exploitation of the information of ordinal relationships among age labels. In their training phase, for a given age label, samples belonging to their neighbouring age labels are also involved; in other words, the weight of each sample in computing the quantities of a specific age label was assigned according to label similarity. However, these two approaches only treat one facial image instance with a single age label (not multiple age labels); as mentioned above, almost all age estimation algorithms also suffer from insufficient training datasets. Inspired by these defects, Geng et al. [21,22] proposed the Label Distribution Learning (LDL) for facial age estimation. This method takes full advantages of the similarities between the authentic age and its adjacent ages instead of regarding each age label as one isolated entity; that is to say, this method better reveals the nature of the human aging process than other existing age estimation algorithms.

**Figure 1:**
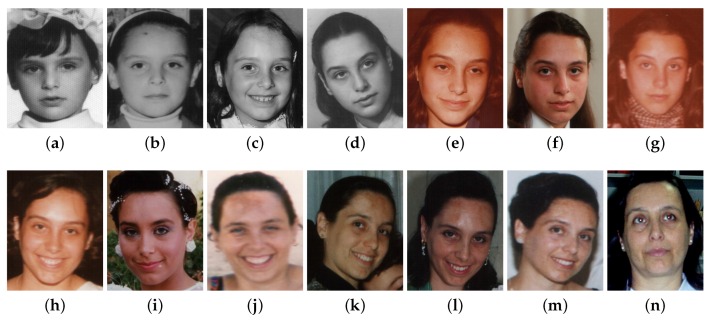
Facial image examples of one subject in sequential order of different age values in the FG-NET database [23]. (**a**) 4; (**b**) 5; (**c**) 7; (**d**) 15; (**e**) 16; (**f**) 18; (**g**) 20; (**h**) 21; (**i**) 23; (**j**) 26; (**k**) 29; (**l**) 31; (**m**) 36; (**n**) 38.

**Figure 2:**
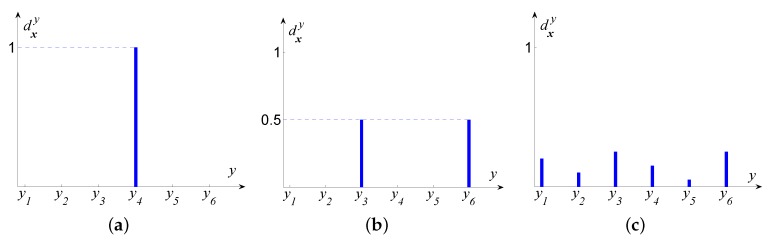
Three different types of label distribution. (**a**) single label; (**b**) multiple label; (**c**) general label distribution.

In detail, Label Distribution Learning (LDL) [21] starts from the observation and intuitive common sense that the faces at adjacent ages tend to have much similarity, which can be shown in Figure 1. An extreme case is that a person’s face would look almost the same on the last day of his or her at 20-years-old and the first day of being a 21-year-old. In other words, adjacent ages can make a great contribution to the chronological (authentic) age. Actually in the real world, we are also more accustomed to judge a person’s age by “about 30-year-old” or “around 40–50” rather than directly telling the exact age. Thus, LDL allocates each facial image an “age label distribution with multi-label” instead of the “chronological age as single-label”. Based on this idea, in order to represent the degree that those adjacent age labels describe this facial image, LDL firstly introduces the concept of *description degree*. Specifically, suppose we have a facial image ***x***, then the *description degree*$d_{x}^{y}\in \left[0,1\right]$ (*y* represents a certain age label in the whole age range) and is the level at which the age *y* depicts this instance. In addition, the description degree must satisfy the restricted condition $\sum_{y}d_{x}^{y}=1$. Figure 2 demonstrates three different types of label distributions for six-class labels. Type (a) shows the most traditionally frequently-used and simplest case: single label. In the figure, the instance is allocated only one label $y_{4}$ and thus $y_{4}$’s description degree $d_{x}^{y_{4}}=1$ while the description degree for $y_{1},y_{2},y_{3},y_{5},y_{6}$ is 0, which means the sole label $y_{4}$ is able to totally describe this instance with other labels contributing nothing. Figure 2b is another case called multiple label, in which multiple labels (two, three or more) would have even description intensities. As can be seen, $y_{3}$ and $y_{6}$ are allocated evenly to describe the instance and each takes up 0.5 (50%) as their description degree, accompanied by other remaining labels contributing nothing with the corresponding description degrees of 0. A more general case is in Figure 2c: each label is allocated a description degree $d_{x}^{y}\in \left[0,1\right]$ with $\sum_{y}d_{x}^{y}=1$.

In general, label distribution is more flexible to represent the ambiguity. However, the learning algorithms from label distribution [21,22] suffer from their simple decision-making criteria which only blindly choose the age label with maximum description degree as the final predicted age and fail to take the characteristics of aging process into account. Actually, in practice, when we finish the step of learning from label distribution and head into the last step of decision-making, it is far from being rare that many age labels would have very similar description degrees. Thus, in this case, rashly assigning the one with the highest description degree would neglect all the other age labels with close description degrees, which may consequently enlarge the final estimation deviation and degrade the algorithm’s overall performance. In this paper, we propose a series of strategic learning algorithms for decision-making to effectively solve this problem in the application of facial age estimation.

The rest of this paper is organized as follows: firstly, the label distribution learning in age estimation and its decision-making criterion is briefly illustrated in Section 2. Then, a series of strategic decision-making learning algorithms for label distribution learning are proposed in Section 3. After that, the experiments and discussion on facial age estimation for different types of age label distribution are reported in Section 4. Finally, Section 5 concludes the paper.

## 2. Label Distribution Learning and Its Decision-Making Criterion

**Figure 3:**
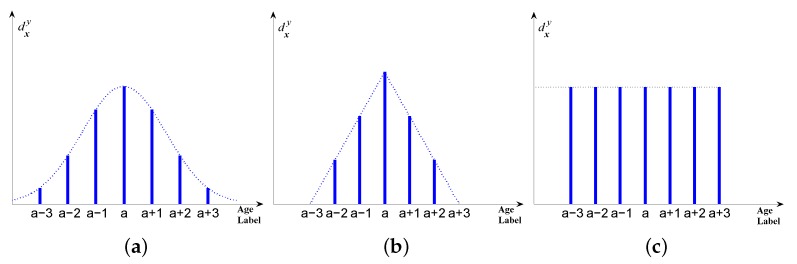
Two primary age label distributions (**a**) Gaussian-like distribution (with seven-class labels); (**b**) triangle distribution (with bottom length of six) and multiple age labels with same description degrees (with seven-class labels) (**c**) for the chronological age **a**.

Figure 3 shows the example of three different types of multiple labels for age representation, in which Figure 3a,b are two primary age label distributions, namely Gaussian-like distribution and triangle distribution; Figure 3c indicates the multiple label with the same description degrees. In the first two distributions, the description degree of the chronological age (authentic age) is the highest; for other age labels on both sides of the chronological age, description degrees decrease symmetrically to the same extent. Particularly, the condition of Figure 3a is not called “Gaussian distribution” but “Gaussian-like distribution” because Gaussian distribution is a continuous function for the independent variable traversing the set of all real numbers. However, the age label is a series of discrete integers with a limited domain. Thus, in the application of age estimation, we only use the shape of Gaussian distribution and discretize the previous “probability density” to constitute description degrees. In detail, firstly calculate pdf(y) for all possible age label *y* (pdf(·) stands for probability density function) and then do the normalization $d_{x}^{y}=pdf\left(y\right)/\sum_{y}pdf(y)$ so that $\sum_{y}d_{x}^{y}=1$ is guaranteed. Note that when generating the label distribution, the description degrees of all age labels are greater than 0 for Gaussian-like distribution (Figure 3a), which means all age labels are involved and contribute to the label distribution; however, in the condition of Figure 3b,c, only part of the age labels contribute to the label distribution (the description degrees of other age labels are 0).

Generally, Label Distribution Learning (LDL) utilizes the methods in statistics to learn a conditional probability mass function from the ready-made label distribution and get the corresponding description degrees [21,22]. In detail, the description degree $d_{x}^{y}$ can be seen mathematically as $d_{x}^{y}=P\left(y|x\right)\in \left[0,1\right]$, which means that, for an instance ***x***, the description degree of the label *y* equals the conditional probability of *y* given ***x***. Next, suppose the input space is denoted by $ℵ={ℜ}^{w}$ and the label set $\Psi =\{y_{1},y_{2},y_{3},...,y_{t}\}$ (*t* is the total number of labels) which contains all involving labels. Then, given a training set with *n* instances $ℑ=\{\left(x_{1},D_{1}\right),\left(x_{2},D_{2}\right),...,\left(x_{n},D_{n}\right)\}$, where $x_{i}\in ℵ$ is the *i*th instance and $D_{i}=\left\{d_{x_{i}}^{y_{1}},d_{x_{i}}^{y_{2}},...,d_{x_{i}}^{y_{t}}\right\}$ is the label distribution for the *i*th instance, the objective is to learn the approximated conditional probability mass function p(y|x) from the training set ℑ, in which x∈ℵ and y∈Ψ.

In order to solve the above-mentioned question, the parameter vector θ needs to be introduced as p(y|x;θ). Then, the problem becomes: to find out a suitable θ that can generate a label distribution approximating $D_{i}$ given $x_{i}$. Then, Kullback–Leibler (KL) divergence is used as the measurement of two distributions’ similarity, which can be represented by:

(1) $$\begin{array}{c} D_{KL}=\sum_{i}\sum_{j}\left(d_{x_{i}}^{y_{j}}ln\frac{d_{x_{i}}^{y_{j}}}{p(y_{j}|x_{i}.\theta )}\right) \end{array}$$

Thus, the optimal solution $\theta_{opt}$ for the parameter vector θ should be obtained by minimizing the KL divergence, namely

(2) $$\begin{array}{cc} \theta_{opt} & =\underset{\theta }{\mathrm{arg min}}\sum_{i}\sum_{j}\left(d_{x_{i}}^{y_{j}}ln\frac{d_{x_{i}}^{y_{j}}}{p(y_{j}|x_{i};\theta )}\right)\\ & =\underset{\theta }{\mathrm{arg max}}\sum_{i}\sum_{j}\left(d_{x_{i}}^{y_{j}}\;ln\;p\left(y_{j}|x_{i};\theta \right)\right) \end{array}$$

Then, p(y|x;θ) can be formulated by maximum entropy model [24] as

(3) $$\begin{array}{c} p\left(y|x;\theta \right)=\frac{1}{\sum_{y}\exp\left(\sum_{k}\theta_{y,k}\tau_{k}\left(x\right)\right)}\exp\left(\sum_{k}\theta_{y,k}\tau_{k}\left(x\right)\right) \end{array}$$

where $\tau_{k}\left(x\right)$ represents the *k*th feature in ***x*** and $\theta_{y,k}$ is one element in the model parameter vector θ. From Equations (2) and (3), the objective function Ω(θ) can be derived as

(4) $$\begin{array}{c} \Omega \left(\theta \right)=\left(\sum_{i,j}d_{x_{i}}^{y_{j}}\sum_{k}\theta_{y_{j},k}\tau_{k}\left(x_{i}\right)\right)-\left(\sum_{i}\ln\sum_{j}\exp\left(\sum_{k}\theta_{y_{j},k}\tau_{k}\left(x_{i}\right)\right)\right) \end{array}$$

IIS(improved iterative scaling)-LLD and BFGS(Broyden-Fletcher-Goldfarb-Shanno)-LLD [21,22] are the main algorithms in dealing with the above optimization problem. After p(y|x;θ) is learned from the training set, the label distribution of any new instance $x^{\prime }$ is $p(y|x^{\prime };\theta )$. Then, the final predicted age is obtained by the following decision-making criterion:

(5) $$\begin{array}{c} y_{final}=\underset{y}{\mathrm{arg max}}\;p\left(y|x^{\prime };\theta \right) \end{array}$$

which can be explained as choosing the age label with the maximum description degree in the calculated label distribution of this new instance.

## 3. Strategic Decision-Making Label Distribution Learning (SDM-LDL) for Facial Age Estimation

So far, three main algorithms based on label distribution learning have been proposed, i.e., IIS-LLD, BFGS-LLD and CPNN (Conditional Probability Neural Network) [21,22]. However, these algorithms seems to put more emphasis on dealing with pure mathematical problems (optimization and parameter tuning) for obtaining the label distribution output p(y|x;θ); on the other hand, they only pick the age label with the highest description degree and neglect the distribution of other labels with similar description degrees which may also contribute much to decision making; in other words, they fail to design more appropriate and complex decision-making criteria specialized for the application of facial age estimation.

Fundamentally, the reason why the decision-making criterion of original LDL is deficient can be explained as follows: the obtained distribution is not symmetrical along two sides of the maximum description degree. In other words, the obtained age label distribution suffers from distributing unevenly with the center of the maximum description degree; specifically, there exists the possibility when the neighboring age labels with relatively high description degrees are located more intensively on one side than the other side. For this condition, the decision-making rule should lean to the abovementioned “more intensive” range/side. That is, more age labels on the “intense” side should be involved in and contribute to the final estimation process than the ones on the “sparse” side (the other side). Consequently, if we still simply pick the highest description degree, then the estimated age will have larger deviations and all of the neighboring high description degrees only second to the maximum value will become meaningless. Inspired by this defect, a series of strategic decision-making algorithms for label distribution learning (SDM-LDL) are proposed for age estimation.

Now suppose that p(y|x;θ) has already been learned from the training set using IIS-LLD, BFGS-LLD or CPNN. Then, the label distribution of a new instance $x^{\prime }$ can be calculated by $p(y|x^{\prime };\theta )$ for all *y* (age labels). In order to obviously compare the differences between these algorithms, both newly proposed algorithms with different decision-making strategies and the original LDL decision-making rule without any strategy are listed below.

### 3.1. Original Decision-Making Rule without Strategy

Scan through all age labels *y* and search for the maximum $p(y|x^{\prime };\theta )$, then the predicted age would be chosen as $y_{p}=\underset{y}{\mathrm{arg max}}\;p\left(y|x^{\prime };\theta \right)$, namely to directly select the age label with the maximum description degree. It is worth noting that, in this method, only one age label gets involved in determining the final predicted age.

### 3.2. Strategic Decision-Making Algorithm (SDM-LDL) with Strategy 1

As mentioned above, the initially obtained age label distribution does not distribute evenly along two sides of the maximum description degree, so merely selecting the age label with the highest description degree will lead to large deviation from the ground truth and suboptimal estimation performance. Consequently, one natural and straightforward idea is to choose multiple age labels with higher description degrees as the “age label base” and the final result (age) can be estimated as the mean value for this “base”. Then, here comes another question: how can the number of the chosen age labels be determined? In Strategy 1, we manually select this value (hereinafter referred to as *N*). The detailed procedure can be summarized as follows. Rank all description degrees in descending order and extract the top *N* description degrees and their corresponding age labels $\left\{y_{s_{1}},y_{s_{2}},...,y_{s_{N}}\right\}$. Then, add these age labels up and obtain the mean value as the final age (note that here *N* is a positive integer in the range 2–10 which is pre-chosen by us and the influence of different values for *N* would be further shown and compared in the experiment section). Thus, in this strategy, the predicted age could be mathematically expressed as

(6) $$\begin{array}{c} y_{p}=\frac{1}{N}\left(y_{s_{1}}+y_{s_{2}}+...+y_{s_{N}}\right) \end{array}$$

This strategy involves *N* age labels in determining the final predicted age.

### 3.3. SDM-LDL with Strategy 2

In Strategy 1, the final estimated age is determined as the mean value of those selected top *N* age labels. In essence, this action evenly (equally) considers all the *N* labels which have different description degrees; in other words, the description degree information for these *N* labels is not utilized. Then, how can we make use of both the above-mentioned description degree information and the top *N* age labels? An effective solution is to calculate the weighted sum, in which the weights are obtained by normalizing the corresponding description degrees of these pre-chosen *N* labels. The concrete steps are as follows. Rank all description degrees in descending order and extract the top *N* description degrees and their corresponding age labels $\left\{y_{s_{1}},y_{s_{2}},...,y_{s_{N}}\right\}$. Then, for these *N* degrees, do the normalization and get the normalized weights. Finally, accumulate the product of the weights and their corresponding age labels. Note that here *N* is also pre-chosen and tested within the range 2–10 as in Strategy 1 in order to see which value in this range would get the best performance and whether the value of *N* would have a regular impact for the final estimation result. In this strategy, the predicted age could be formulated as

(7) $$\begin{array}{c} y_{p}=\sum_{y_{s}=y_{s_{1}}}^{y_{s_{N}}}y_{s}\times \frac{p(y_{s}|x^{\prime };\theta )}{\sum_{y_{s}=y_{s_{1}}}^{y_{s_{N}}}p(y_{s}|x^{\prime };\theta )} \end{array}$$

This strategy involves *N* age labels and their corresponding description degrees in determining the final predicted age.

### 3.4. SDM-LDL with Strategy 3

In Strategies 1 and 2, the number of age labels is manually chosen. Whether this value is properly selected or not will have a direct impact on age estimation performance. Thus, another question comes to our mind: can we find an appropriate adaptive value for the number of chosen age labels so that Strategy 1 can be autonomously conducted without human interference? Driven by this question, we focus on the differences between every two adjacent description degrees in descending order: to a great extent, the largest difference is an indicator to distinguish the age labels with higher description degrees from those with lower description degrees. The whole process can be described as follows. Rank all description degrees in descending order $\left\{y_{s_{1}},y_{s_{2}},...,y_{s_{t}}\right\}$ (*t* is the total number of age labels), calculate the differences between adjacent description degrees and obtain the set $\left\{di_{1},di_{2},...,di_{t-1}\right\}$, where $di_{1}=y_{s_{1}}-y_{s_{2}}$, $di_{2}=y_{s_{2}}-y_{s_{3}}$, ..., $di_{t-1}=y_{s_{t-1}}-y_{s_{t}}$. Then, find the maximum di (denoted by $di_{G}$; in other words, the sequence number of this maximum di is denoted as *G*) and calculate the mean value from the top *G* description degrees in descending order, namely $\left\{y_{s_{1}},y_{s_{2}},...,y_{s_{G}}\right\}$. Consequently, the predicted age could be described as

(8) $$\begin{array}{c} y_{p}=\frac{1}{G}\left(y_{s_{1}}+y_{s_{2}}+...+y_{s_{G}}\right) \end{array}$$

In this strategy, *G* age labels directly contribute to the final determination of the predicted age.

### 3.5. SDM-LDL with Strategy 4

Inspired by Strategies 2 and 3, we are motivated to combine the advantages of these two methods. Firstly, the proper *G* is obtained autonomously, then the normalization and weighted sum are conducted to use both the age label and the corresponding description degree information. The specific procedure is summarized as follows. Rank all description degrees in descending order $\left\{y_{s_{1}},y_{s_{2}},...,y_{s_{t}}\right\}$ (*t* is the total number of age labels), calculate the differences between adjacent description degrees and obtain the set $\left\{di_{1},di_{2},...,di_{t-1}\right\}$, where $di_{1}=y_{s_{1}}-y_{s_{2}}$, $di_{2}=y_{s_{2}}-y_{s_{3}}$, ..., $di_{t-1}=y_{s_{t-1}}-y_{s_{t}}$. Then, find the maximum di (denoted by $di_{G}$); for these G degrees, do the normalization and get the normalized weights. Finally, accumulate the product of the weights and their corresponding age labels. Consequently, the predicted age could be described as

(9) $$\begin{array}{c} y_{p}=\sum_{y_{s}=y_{s_{1}}}^{y_{s_{G}}}y_{s}\times \frac{p(y_{s}|x^{\prime };\theta )}{\sum_{y_{s}=y_{s_{1}}}^{y_{s_{G}}}p(y_{s}|x^{\prime };\theta )} \end{array}$$

In this strategy, *G* age labels and their corresponding description degrees directly contribute to the final determination of the predicted age.

It is worth mentioning that Strategies 3 and 4 utilize successive differences to seek out a specific boundary distinguishing between the age labels with great contribution and small contribution, so that the age labels with great contribution would be adopted for the final predicted age and those with small contribution would be discarded.

### 3.6. SDM-LDL with Strategy 5

Strategies 1–4 adopt only partial age labels so that the complete description degrees are not fully exploited (only part of description degrees involved). Thus, we design this strategy to take advantage of all age labels and all the description degrees: multiply age labels by their corresponding description degrees respectively and adopt the cumulative value of these product as the final result. Then, the predicted age would be calculated by

(10) $$\begin{array}{c} y_{p}=\sum_{y=y_{1}}^{y_{t}}y\times p(y|x^{\prime };\theta ) \end{array}$$

**Figure 4:**
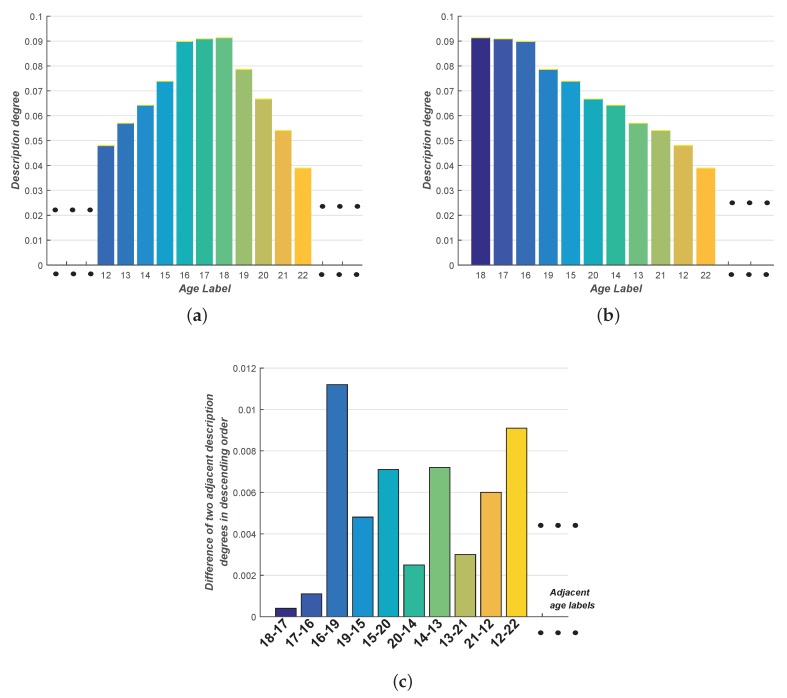
Histogram illustrations for the example of Table 1 and Table 2: (**a**) corresponds to Table 1; (**b**) and (**c**) correspond to Table 2.

For a more distinct demonstration, we take an example for further illustration, as is shown in Table 1 and Table 2 and Figure 4. Suppose we have already learned p(y|x;θ) from the training set using IIS-LLD, BFGS-LLD or CPNN. Now when a new facial image $x^{\prime }$ comes in, the corresponding age label distribution can be obtained as in Table 1 and Figure 4a. Then, on the basis of Table 1, we rank all these description degrees in descending order and also calculate the successive differences di one by one, which is shown in Table 2 and Figure 4b,c. Next, different types of age are listed as follows:

Table 3 shows the comparison of deviation from the authentic age 16 using the proposed SDM-LDLs with all strategies as well as the original LDL. As can be seen from the table, all the SDM-LDLs achieve smaller deviation compared to the original LDL without any decision-making strategy. Although it is only a possible example of the obtained age label distribution, this illustration vividly shows the deficiency of original LDL’s decision-making criterion due to the obtained distribution’s asymmetry along two sides of the maximum description degree. On the contrary, the proposed SDM-LDL with various strategies is specially designed to suit the inner characteristics of aging and also make up for this drawback, which takes full advantage of the whole age label distribution and offsets the deficiency and deviations caused by the original LDL’s decision-making rule to the greatest extent. As for the above example, the relatively high description degrees occur more frequently on the left side of the maximum description degree (18 with the description degree of 0.0913) than on the right side (15, 16, 17 on the left side versus 19 on the right side). Thus, the predicted age should lean to the direction of “smaller than 18” rather than 18, as obtained from the five strategies of SDM-LDL.

## 4. Experiments

### 4.1. Experimental Environment Settings

The aging database that our experiments are conducted on is the most popular facial aging benchmark: FG-NET [23]. FG-NET has 1002 grayscale or color facial images of 82 people, which includes comprehensive poses, expressions and lighting environments. Just as Table 4 shows, all of the people’s age ranges are from 0 to 69, with the young and middle-aged taking up the majority and the proportion of old people much smaller. In order to uniformly processing, all facial images in FG-NET are converted to grayscale, aligned and normalized. Finally histogram equalization is conducted in order to decrease the illumination influence.

In order to increase the accuracy of the final predicted age, three feature models for information extraction were used from FG-NET raw images: Active Appearance Model (AAM) [25], local binary patterns (LBP) and Bio-inspired feature (BIF) [26], which would be combined together for a total dataset. Active Appearance Models (AAM) can represent both shape and texture information instead of only facial geometry, which is also popularly selected by other age estimation methods. LBP is also a widely-used feature for texture classification in computer vision. BIF was selected because of its high age estimation accuracy. The information extracted from the above three feature models can complement each other; together, they were combined as a total dataset. For AAM features, the feature dimension was set to retain 95% of variability. For BIF features, the number of bands was set at eight (16 scales totally) with four orientations each. In addition, to reduce the entire feature space, principal component analysis (PCA) was used to reduce the dimension. More specifically, all three of the feature models would be reduced to 100 dimensions, respectively. Particularly, the AAM model includes both shape and texture information, so these two sub-properties would be reduced to 50 dimensions respectively (in total 100 dimensions). Furthermore, leave-one-person-out (LOPO), a popular test procedure, was utilized for the test strategy, which was suggested in [5,10,19,27,28].

### 4.2. Methodology and Experimental Results

In age estimation, the most popular performance measurement is the mean absolute error (MAE), which can be described by

(11) $$\begin{array}{c} MAE=\sum_{m=1}^{T}\left|{y_{m}}^{*}-y_{m}\right|/T \end{array}$$

where ${y_{m}}^{*}$ is the estimated age, $y_{m}$ is the authentic age and T is the number of test images.

Just as mentioned above, when initially generating the age label distribution for a given chronological age, there are three different ways, i.e., Gaussian-like distribution, triangle distribution and multi-label distribution with equal description degrees (Figure 3a–c). These three conditions have their respective features and thus the final predicted ages based on them are different from each other. Consequently, these three conditions will firstly be analyzed separately and then compared with each other to obtain the overall conclusion. Note that our proposed algorithm is applicable to all existing LDL methods (IIS-LLD, BFGS-LLD, CPNN); however, for all the following LDL-based experiments (including original LDL and the proposed SDM-LDL), we only utilize the BFGS-LLD method in computing p(y|x;θ) to maintain consistency.

#### 4.2.1. Gaussian-Like Distribution

First of all, when generating the Gaussian-like age label distribution, the controlled variable is the standard deviation *σ*. Thus, in the following experiment of this section, when realizing every strategy mentioned above, we will assign an integer range 1–10 to the standard deviation and make comparisons of the final predicted age with different standard deviations. Note that for the standard deviation *σ*, the integer range 1–10 is quite a broad range because for σ=5,6,7 and even greater, the age label distribution tends to become “flatter and flatter”, which means description degrees of different age labels would get closer to the description degree of the actual age and the description degrees’ disparities among different age labels become less and less obvious. However, the standard deviation is given a great range in order to more clearly display the rule and tendency of the standard deviation’s impact on the overall estimation performance.

**Figure 5:**
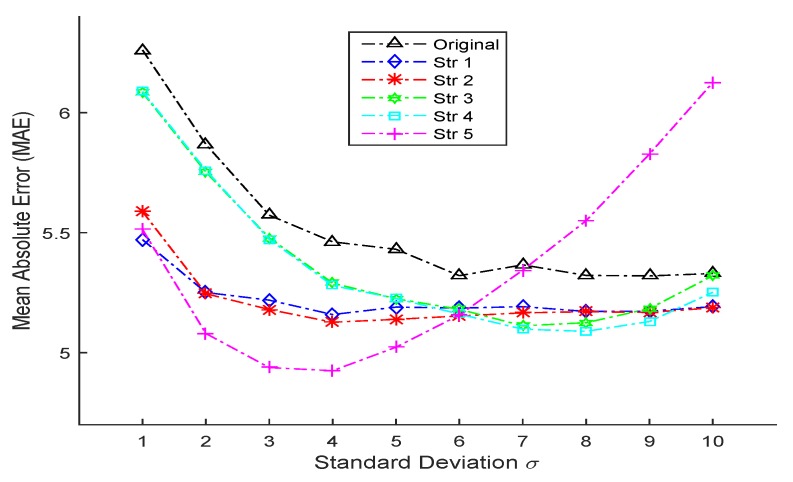
The line chart (variation tendency) of MAEs with respect to different standard deviations for the original LDL algorithm and the proposed SDM-LDL algorithms with different strategies when age label distribution is generated as **Gaussian-like**.

Table 5 shows the comprehensive comparisons including different strategies used in the proposed SDM-LDL algorithms and different standard deviations *σ* when age label distribution is generated as Gaussian-like. In the vertical direction, for every value of *σ*, almost all of the SDM-LDL algorithms with different strategies obtain smaller MAE than the original LDL algorithm, which demonstrate the validity and superiority of the proposed algorithms with all strategies. In addition, when *σ* varies, different strategies show their respective advantages. For example, when σ=1, Strategy 1 gets the best performance; when σ=2,6,10, Strategy 2 gets the optimal results; when σ=7,8,9, Strategy 4 gets the smallest MAEs and when σ=3,4,5, Strategy 5 outperforms all other strategies. Horizontally, when *σ* increases from one to 10, MAEs of all LDL-based algorithms (including original LDL and SDM-LDL) exhibit the general tendency of first decreasing and then increasing, which indicates that there exists an optimal value (or a small range) of *σ* to suit different algorithms and for different algorithms, such an optimal value varies. For instance, if using SDM-LDL with Strategy 5, then the optimal value for *σ* is four; however in the utilization of SDM-LDL with Strategy 3, the optimal value for *σ* is seven. More intuitionally from Figure 5, most parts of the fold lines of Strategy 1–5 fall below that of the original LDL algorithm and this means our proposed algorithms get superior accuracy compared to the original one. In addition, in general, almost all fold lines in Figure 5 follow the tendency of first going down and then going up. Moreover, the fold line located in the bottom is changing with the variation of *σ*, indicating the best strategy varies for different standard deviations. Noticing that for Strategy 5, when *σ* gets bigger, the MAE firstly drops quickly and then rises dramatically, so the appropriate *σ* value for Strategy 5 can be selected from 2,3,4,5. In fact, the reason why the Strategy 5 fold line drastically climbs afterwards is obvious: when *σ* becomes very large and extends a certain range, the description degrees for all age labels will get very close. In this case, when we accumulate all the products of age labels and their corresponding description degrees according to Strategy 5, the final obtained age would approach the median of the whole age label range, which, as a result, makes the deviation bigger and bigger. Just imagine in extreme cases when *σ* approaches to infinity so that the description degrees of all age labels are equivalent, then if using Strategy 5, the calculated age result will be the median value in the age label range. In particular, for the most commonly used standard deviation *σ* ranging from 1 to 5, the fold line of the original LDL falls steeply and does not tend to become stable, which means the original LDL is not robust enough for typical values of standard deviation. In addition, in this range of *σ*, the original LDL does not reach the optimal MAE. In contrast, the proposed SDM-LDL with Strategies 1,2,5 all reach the lowest value of MAE for σ∈[1,5] with big advantages over the “Original” fold line as well as more robustness. In addition, chances are greater that one is going to use the LDL-based algorithms in facial age estimation and does not have so much time to conduct a series of trials seeking for the optimal standard deviation: he or she only chooses from the most commonly used values 1–5. In this case, our proposed SDM-LDL with Strategies 1,2,5 are more likely to obtain optimal results or the results approaching the optimal MAE. Furthermore, the SDM-LDL with Strategies 1 and 2 shows robustness with their more stable and flatter fold lines as evidence: throughout the range σ∈[1,10], the MAE of Strategies 1 and 2 always remains a relatively low value with no sharp fluctuations. Note that for SDM-LDL with Strategies 1 and 2, only the best results are presented in the table along with the value of *N* at that time, so one natural question comes: when *N* traverses from two to 10, what will the variation trend of the MAE be, or how does the value of *N* influence the estimation performance?

Table 6 shows the results of different Ns (from 2 to 10) impacts on MAEs using the proposed SDM-LDL algorithms with Strategies 1 and 2. As can be seen, for both Strategies 1 and 2, the MAE generally tends to be smaller when *N* is bigger, no matter what value of *σ* is. For example, for Strategy 2, when *N* takes the value of relatively big integers, like 7, 9 and 10, the majority of results are optimal whatever *σ* is. Therefore, normally taking a value greater than 5 for *N* will get better performance.

#### 4.2.2. Triangle Distribution

Just as Figure 3b shows, unlike Gaussian-like distribution, triangle distribution only takes advantage of partial age labels that are located on both sides near the chronological age; in other words, it only allocates the description degree to the “neighboring" age labels of the authentic age while description degrees of other age labels remain 0. Furthermore, the description degree reaches the peak value at the chronological age, of which it drops linearly and symmetrically on both sides.

**Figure 6:**
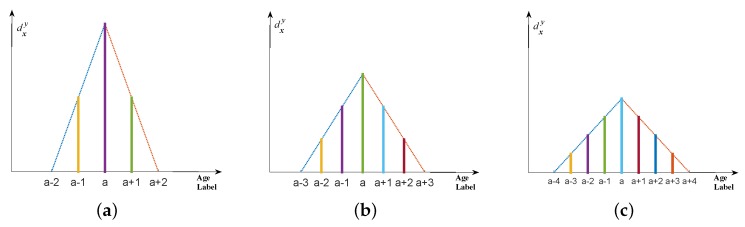
Different bottom lengths when generating the **triangle** age label distribution for the chronological age **a**. (**a**) bottom length of 4; (**b**) bottom length of 6; (**c**) bottom length of 8.

When generating the triangle age label distribution, the controlled variable is the bottom length. Figure 6 demonstrates three examples of different bottom lengths, i.e., 4, 6 and 8. As can be seen from this figure, when the bottom length becomes greater, the description degree of the chronological age is smaller and the differences between the chronological age and other neighboring age labels are smaller. Intuitively from the “shape”, the triangle becomes flatter with the increase of the bottom length (from Figure 6a to b,c). In experiments, the bottom length is given a broad value range of {2,4,6,...,30} to examine its impact and variation tendency for the estimated age.

**Figure 7:**
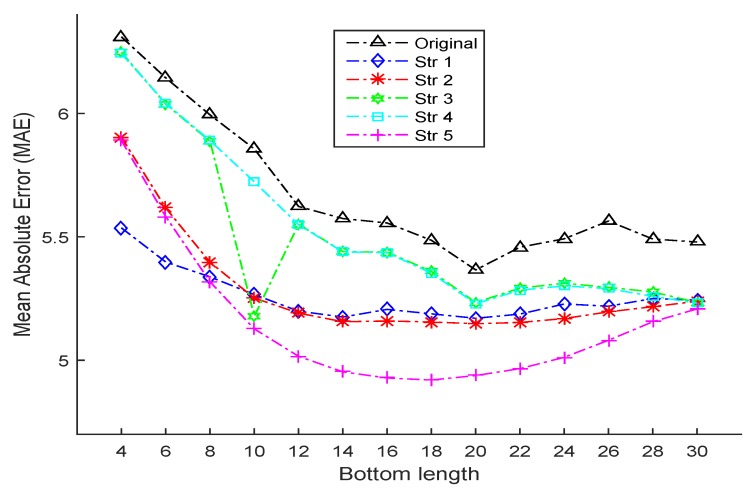
The line chart (variation tendency) of MAEs with respect to different bottom lengths for the original LDL algorithm and the proposed SDM-LDL algorithms with different strategies when age label distribution is generated as **triangle**.

Table 7 shows the MAE results with different bottom lengths (from 4 to 30) using the original LDL and the proposed SDM-LDL algorithms with all strategies. As can be seen clearly, when firstly generating the age label distribution as triangle, the proposed SDM-LDL algorithm with Strategy 5 almost outperforms all the other strategies, especially when the bottom length is not very small; in addition, similar to the conclusion from Gaussian-like age label distribution, vertically for every value of the bottom length, MAEs of all the proposed strategies are smaller without exception, indicating the validity and superiority of the proposed SDM-LDL in the case of triangle distribution. On the other hand, Figure 7 can also be used as a corroboration of this conclusion: for almost all the ranges of the bottom length, the fold line of Strategy 5 remains the lowest except from 4 to 8, where it only lags behind the fold line of Strategy 1. Moreover, the proposed SDM-LDL algorithms with all strategies are superior to the original LDL algorithm with all fold lines falling below the original line. As for the triangle age label distribution, when the bottom length becomes greater, the MAE also has the trend of firstly decreasing sharply and then increasing slightly (or becoming stable). Moreover, the SDM-LDL algorithms are more robust than the original LDL, especially for Strategies 1 and 2.

Table 8 shows the variation trends of MAEs with *N* from 2 to 10 using Strategies 1 and 2 of the proposed SDM-LDL on the condition that age label distribution is generated as a triangle. For both Strategies 1 and 2, small values of *N* (2–6) yield inferior performance against bigger values (7–10). Particularly for Strategy 2, whatever the bottom length is, the best performance always happens when *N* equals 9 and 10. As a conclusion, for Strategies 1 and 2, if the age label distribution is initially generated as the triangle style, then it is better to allocate *N* a relatively big value (7–10) for better estimation performance, especially for Strategy 2, where *N* should be given the value of 9 and 10.

#### 4.2.3. Multi-Label Distribution with Equal Description Degrees

**Figure 8:**
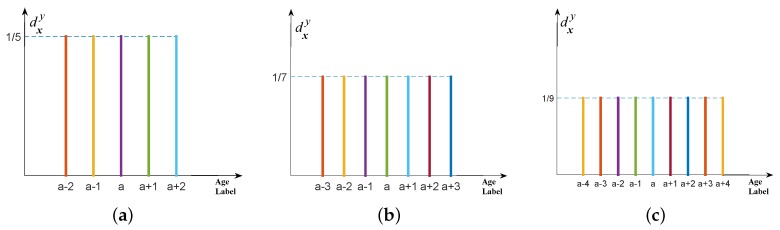
Different number of age labels when generating age label distribution as **multi-label with equal description degrees** for the chronological age *a*. (**a**) number of age labels: 5; (**b**) number of age labels: 7; (**c**) number of age labels: 9.

As Figure 3c shows, in this situation, description degrees are evenly distributed to the chronological age and the adjacent age labels. Thus, when initially generating age label distribution in this style, the controlled variable is the number of age labels. Figure 8 shows the example of different number of age labels: if the number of age labels is 5 and 7, then the description degrees for all involved age labels are 1/5 and 1/7, respectively. In other words, when the number of age labels increases, the description degrees for all involved age labels will reduce accordingly. In experiments, to explore its influence for the estimation performance, the number of age labels is allocated as 3,5,7,...,15.

**Figure 9:**
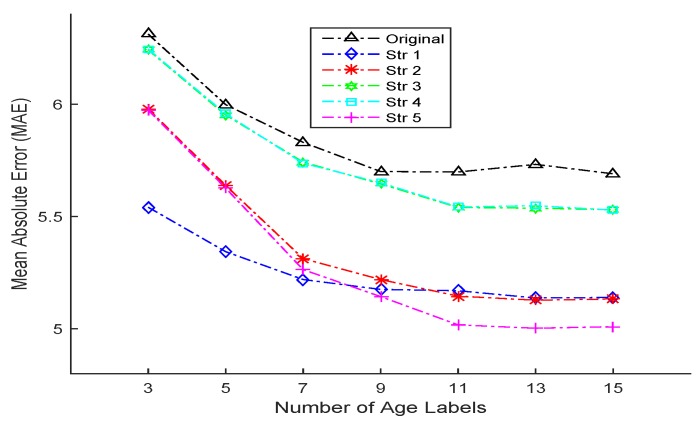
The line chart (variation tendency) of MAEs with respect to different number of age labels for the original LDL and the proposed SDM-LDL with different strategies when age label distribution is generated as **multi-label with equal description degrees**.

Table 9 demonstrates the estimation performance of all compared LDL-based algorithms with different number of age labels (from three to 15) when initially generating age label distribution as multi-label with equal description degrees. Again, all the proposed methods outcompete the existing original LDL algorithm. In addition, when the number of age labels is small (3–7), Strategy 1 obtains the best results; however, as the number of age labels gets bigger (9–15), Strategy 5 shows advantages over the others in performance. Figure 9 also supports this argument: when the number of age labels are small (3–7), the lowest fold line is Strategy 1; when the coordinates of the horizontal axes are larger, the lowest line becomes Strategy 5. In the end, the fold lines of all methods tend to be stable. In addition, Strategy 1 shows more robustness with the evidence of remaining the relatively low MAE for a different number of age labels.

As for different *N* impacts on the final estimated age for Strategies 1 and 2, the conclusion is the same as in the “Triangle distribution" part. Thus, the detailed experimental data are omitted for simplicity.

#### 4.2.4. Overall comparison of the Proposed SDM-LDL Algorithms and Other Popular Algorithms

The preceding parts discussed the respective estimation results in detail when age label distribution is initially generated by three different patterns, namely Gaussian-like, triangle and multi-label with equal description degrees. In the following, these three patterns are compared as a whole to see which one is the best choice when generating the age label distribution.

As can be apparently seen from Table 10, for almost all LDL-based methods, including original LDL and the proposed SDM-LDL with different strategies, generating Gaussian-like age label distribution yields highest precision and thus achieves best performance, followed by triangle distribution; the worst choice is using multi-label distribution with equal description degrees.

Table 11 demonstrates the performance of proposed SDM-LDL algorithm with different strategies compared with other existing popular facial age estimation algorithms [5,10,18,26,29,30,31,32,33,34,35,36,37] and the conventional single classification methods Support Vector Machine (SVM) and k-Nearest Neighbors (kNN). From the table, our proposed SDM-LDL with all strategies outperforms the original LDL; when compared with other existing popular methods, SDM-LDL can also achieve relatively good or even superior performance, which proves SDM-LDL’s validity and advantages.

## 5. Conclusions

This paper proposes a novel strategic decision-making algorithm with a series of strategies for label distribution learning in facial age estimation. All strategies are specially designed to suit the characteristics of aging problem. Comprehensive experiments for three different kinds of age label distribution (Gaussian-like, triangle and multi-label with equal description degrees) prove the validity, superiority and robustness of the proposed SDM-LDL algorithms against the original LDL and other existing facial age estimation algorithms. In addition, the respective advantages and properties for each strategy in SDM-LDL are summarized. Further experiments discover the performance’s variation tendencies within each kind of age label distribution so that the most suitable values (or ranges) of those uncertain variables are obtained.

In future work, we plan to expand our approach to one or two more larger-scale aging databases, such as the MORPH Album 2 and FRGC databases, which contain many more facial images and different age structures.

## Figures and Tables

**Table 1:** An example of the obtained age label distribution for a new facial image $x^{\prime }$ (with the authentic age of 16).

Age Label	0	1	2	...	12	13	14	15	16
Description degree	0.0010	0.0017	0.0026	...	0.0480	0.0570	0.0642	0.0738	0.0898
Age Label	17	18	19	20	21	22	...	68	69
Description degree	0.0909	0.0913	0.0786	0.0667	0.0540	0.0389	...	1.0667 $\times 10^{-5}$	2.7528 $\times 10^{-5}$

**Table 2:** Based on Table 1, the age label distribution with descending-order description degrees and successive differences di.

Age Label	18	17	16	19	15	20	14	13	21	12	22	...
Description degree (descending order)	0.0913	0.0909	0.0898	0.0786	0.0738	0.0667	0.0642	0.0570	0.0540	0.0480	0.0389	...
Difference (di)	4e-04	0.0011	0.0112	0.0048	0.0071	0.0025	0.0072	0.0030	0.0060	0.0091	...	...

**Table 3:** The comparison of deviations from authentic age based on the example of Table 1.

	Deviation from Authentic Age (Absolute Value)
Original LDL	2.0000
SDM-LDL (Str 1)	0.5000
SDM-LDL (Str 2)	0.6732
SDM-LDL (Str 3)	1.0000
SDM-LDL (Str 4)	1.0055
SDM-LDL (Str 5)	0.2586

**Table 4:** FG-NET’s age level distribution.

Range of Age	FG-NET	
	#img.	%
0–9	371	37.03
10–19	339	33.83
20–29	144	14.37
30–39	79	7.88
40–49	46	4.59
50–59	15	1.50
60–69	8	0.80
Total	1002	100

**Table 5:** Mean Absolute Errors (MAEs) of the compared original LDL algorithm without decision-making strategy and the proposed SDM-LDL algorithms with different strategies on the condition that age label distribution is generated as **Gaussian-like** (*σ* from 1 to 10; for Strategies 1 and 2, best results are shown in the table along with the value of *N* then; for every *σ*, the optimal MAEs are marked in bold).

***σ***	**1**	**2**	**3**	**4**	**5**
MAE (Ori)	6.261	5.868	5.573	5.462	5.431
MAE (Str 1)	**5.471** (*N* = 8)	5.251 (*N* = 9)	5.219 (*N* = 9)	5.159 (*N* = 9)	5.190 (*N* = 9)
MAE (Str 2)	5.588 (*N* = 10)	5.246 (*N* = 10)	5.180 (*N* = 10)	5.127 (*N* = 10)	5.139 (*N* = 10)
MAE (Str 3)	6.084	5.753	5.478	5.290	5.225
MAE (Str 4)	6.088	5.760	5.473	5.280	5.226
MAE (Str 5)	5.513	**5.080**	**4.938**	**4.920**	**5.023**
***σ***	**6**	**7**	**8**	**9**	**10**
MAE (Ori)	5.321	5.366	5.322	5.320	5.330
MAE (Str 1)	5.186 (*N* = 9)	5.192 (*N* = 9)	5.172 (*N* = 5)	5.173 (*N* = 7)	5.192 (*N* = 7)
MAE (Str 2)	**5.153** (*N* = 9)	5.166 (*N* = 9)	5.171 (*N* = 5)	5.168 (*N* = 7)	**5.187** (*N* = 7)
MAE (Str 3)	5.181	5.112	5.125	5.184	5.321
MAE (Str 4)	5.161	**5.098**	**5.089**	**5.130**	5.252
MAE (Str 5)	5.157	5.344	5.550	5.828	6.126

**Table 6:** The impacts of different values of *N* (2–10) on MAEs using the proposed SDM-LDL algorithms with Strategies 1 and 2 when age label distribution is generated as **Gaussian-like** (*σ* from one to 10; for every *σ*, the optimal MAEs are marked in bold).

*σ*		1	2	3	4	5	6	7	8	9	10
MAE (Str 1)	*N* = 2	5.978	5.725	5.500	5.384	5.414	5.286	5.339	5.264	5.279	5.278
	*N* = 3	5.741	5.592	5.397	5.327	5.358	5.275	5.303	5.221	5.237	5.226
	*N* = 4	5.569	5.528	5.362	5.301	5.339	5.258	5.306	5.205	5.222	5.229
	*N* = 5	5.508	5.401	5.335	5.252	5.263	5.233	5.248	**5.172**	5.193	5.201
	*N* = 6	5.484	5.362	5.286	5.226	5.278	5.206	5.246	5.196	5.196	5.208
	*N* = 7	5.511	5.307	5.233	5.195	5.219	5.189	5.209	5.181	**5.173**	**5.192**
	*N* = 8	**5.471**	5.285	5.245	5.200	5.224	5.199	5.211	5.213	5.188	5.254
	*N* = 9	5.531	**5.251**	**5.219**	**5.159**	**5.190**	**5.186**	**5.192**	5.207	5.189	5.223
	*N* = 10	5.627	5.263	5.241	5.196	5.205	5.241	5.245	5.260	5.226	5.305
MAE (Str 2)	*N* = 2	6.025	5.741	5.501	5.385	5.413	5.287	5.339	5.265	5.280	5.278
	*N* = 3	5.870	5.623	5.408	5.328	5.359	5.274	5.303	5.222	5.238	5.228
	*N* = 4	5.782	5.568	5.373	5.300	5.337	5.257	5.303	5.203	5.221	5.229
	*N* = 5	5.724	5.473	5.343	5.256	5.266	5.233	5.247	**5.171**	5.191	5.200
	*N* = 6	5.689	5.422	5.296	5.223	5.270	5.199	5.238	5.191	5.191	5.204
	*N* = 7	5.647	5.367	5.251	5.194	5.215	5.181	5.201	5.174	**5.168**	**5.187**
	*N* = 8	5.621	5.323	5.224	5.180	5.200	5.175	5.192	5.195	5.175	5.240
	*N* = 9	5.599	5.284	5.203	5.143	5.161	**5.153**	**5.166**	5.185	5.171	5.208
	*N* = 10	**5.588**	**5.246**	**5.180**	**5.127**	**5.139**	5.170	5.189	5.216	5.193	5.273

**Table 7:** MAEs of the compared original LDL algorithm without decision-making strategy and the proposed SDM-LDL algorithms with different strategies on the condition that age label distribution are generated as **triangle** (bottom lengths from 4 to 30; for Strategies 1 and 2, best results are shown in the table along with the value of *N* then; for every bottom length, the optimal MAEs are marked in bold).

**Bottom Length**	**4**	**6**	**8**	**10**	**12**	**14**	**16**
MAE (Ori)	6.310	6.144	5.994	5.856	5.623	5.574	5.556
MAE (Str 1)	**5.536** (*N* = 7)	**5.398** (*N* = 7)	5.338 (*N* = 8)	5.266 (*N* = 7)	5.199 (*N* = 9)	5.175 (*N* = 10)	5.206 (*N* = 10)
MAE (Str 2)	5.903 (*N* = 10)	5.617 (*N* = 10)	5.397 (*N* = 10)	5.252 (*N* = 10)	5.191 (*N* = 10)	5.156 (*N* = 10)	5.159 (*N* = 10)
MAE (Str 3)	6.247	6.038	5.887	5.178	5.551	5.440	5.438
MAE (Str 4)	6.242	6.041	5.890	5.723	5.549	5.439	5.436
MAE (Str 5)	5.890	5.582	**5.318**	**5.127**	**5.016**	**4.953**	**4.928**
**Bottom Length**	**18**	**20**	**22**	**24**	**26**	**28**	**30**
MAE (Ori)	5.486	5.366	5.458	5.492	5.564	5.491	5.479
MAE (Str 1)	5.188 (*N* = 9)	5.170 (*N* = 9)	5.187 (*N* = 9)	5.228 (*N* = 7)	5.219 (*N* = 9)	5.250 (*N* = 9)	5.244 (*N* = 7)
MAE (Str 2)	5.155 (*N* = 10)	5.148 (*N* = 9)	5.153 (*N* = 10)	5.169 (*N* = 10)	5.196 (*N* = 10)	5.217 (*N* = 9)	5.239 (*N* = 9)
MAE (Str 3)	5.360	5.234	5.292	5.312	5.295	5.277	5.234
MAE (Str 4)	5.353	5.229	5.284	5.301	5.290	5.259	5.231
MAE (Str 5)	**4.925**	**4.938**	**4.966**	**5.012**	**5.081**	**5.156**	**5.208**

**Table 8:** The impacts of different values of *N* (2–10) on MAEs using the proposed SDM-LDL algorithms with Strategies 1 and 2 when age label distribution is generated as **triangle** (bottom lengths from 4 to 30; for every value of bottom length, the optimal MAEs are marked in bold).

Bottom Length		4	6	8	10	12	14	16	18	20	22	24	26	28	30
MAE (Str 1)	*N* = 2	5.861	5.802	5.833	5.640	5.549	5.486	5.477	5.453	5.373	5.445	5.438	5.444	5.414	5.436
	*N* = 3	5.656	5.646	5.692	5.524	5.456	5.402	5.402	5.385	5.358	5.395	5.398	5.409	5.364	5.403
	*N* = 4	5.640	5.578	5.523	5.449	5.421	5.309	5.384	5.374	5.326	5.375	5.351	5.382	5.365	5.369
	*N* = 5	5.553	5.463	5.444	5.338	5.324	5.302	5.300	5.293	5.247	5.289	5.276	5.304	5.324	5.326
	*N* = 6	5.542	5.423	5.388	5.323	5.260	5.269	5.309	5.261	5.258	5.250	5.245	5.305	5.314	5.313
	*N* = 7	**5.536**	**5.398**	5.341	**5.266**	5.217	5.229	5.258	5.248	5.207	5.199	**5.228**	5.250	5.261	**5.244**
	*N* = 8	5.673	5.438	**5.338**	5.276	5.221	5.208	5.234	5.227	5.201	5.209	5.230	5.258	5.258	5.259
	*N* = 9	5.774	5.437	5.344	5.275	**5.199**	5.181	5.237	**5.188**	**5.170**	**5.187**	5.234	**5.219**	**5.250**	5.268
	*N* = 10	5.845	5.485	5.386	5.307	5.231	**5.175**	**5.206**	5.211	5.222	5.215	5.235	5.259	5.314	5.300
MAE (Str 2)	*N* = 2	6.071	5.888	5.838	5.663	5.554	5.485	5.483	5.451	5.371	5.443	5.439	5.444	5.416	5.434
	*N* = 3	5.984	5.793	5.729	5.567	5.481	5.417	5.414	5.388	5.357	5.396	5.398	5.410	5.367	5.402
	*N* = 4	5.964	5.743	5.634	5.496	5.456	5.339	5.391	5.374	5.319	5.375	5.353	5.383	5.366	5.368
	*N* = 5	5.941	5.706	5.562	5.431	5.384	5.332	5.321	5.301	5.244	5.296	5.283	5.312	5.327	5.327
	*N* = 6	5.929	5.673	5.496	5.370	5.330	5.294	5.313	5.265	5.246	5.251	5.244	5.301	5.308	5.309
	*N* = 7	5.919	5.648	5.457	5.334	5.289	5.263	5.270	5.250	5.207	5.202	5.223	5.247	5.256	5.242
	*N* = 8	5.912	5.640	5.438	5.309	5.252	5.229	5.229	5.218	5.184	5.194	5.207	5.236	5.237	5.241
	*N* = 9	5.908	5.629	5.416	5.280	5.221	5.193	5.208	5.176	**5.148**	5.166	5.199	5.199	**5.217**	**5.239**
	*N* = 10	**5.903**	**5.617**	**5.397**	**5.252**	**5.191**	**5.156**	**5.159**	**5.155**	5.151	**5.153**	**5.169**	**5.196**	5.242	5.243

**Table 9:** MAEs of the compared original LDL algorithm without decision-making strategy and the proposed SDM-LDL algorithms with different strategies on the condition that age label distribution is generated as **multi-label with equal description degrees** (number of age labels from 3, 5, 7, ... to 15; for Strategies 1 and 2, best results are shown in the table along with the value of *N* then; for every number of age labels, the optimal MAEs are marked in bold).

Number of Labels	3	5	7	9	11	13	15
MAE (Ori)	6.312	5.998	5.830	5.700	5.698	5.732	5.690
MAE (Str 1)	**5.541** (*N* = 5)	**5.343** (*N* = 8)	**5.219** (*N* = 7)	5.175 (*N* = 8)	5.169 (*N* = 7)	5.137 (*N* = 9)	5.139 (*N* = 9)
MAE (Str 2)	5.979 (*N* = 10)	5.638 (*N* = 10)	5.312 (*N* = 10)	5.219 (*N* = 10)	5.145 (*N* = 10)	5.127 (*N* = 10)	5.132 (*N* = 10)
MAE (Str 3)	6.242	5.952	5.742	5.645	5.540	5.537	5.530
MAE (Str 4)	6.245	5.957	5.737	5.650	5.542	5.548	5.528
MAE (Str 5)	5.974	5.626	5.263	**5.143**	**5.017**	**5.002**	**5.009**

**Table 10:** MAEs of the compared three different patterns in generating age label distribution (the controlled variables in each pattern are traversed within the whole given range and best results are reported; for every row in the table, the optimal MAEs are marked in bold).

	Gaussian-Like	Triangle	Multi-Label with Equal Description Degrees
MAE (Ori)	**5.320**	5.366	5.69
MAE (Str 1)	5.159	5.170	**5.137**
MAE (Str 2)	**5.127**	5.148	5.127
MAE (Str 3)	**5.112**	5.178	5.530
MAE (Str 4)	**5.089**	5.229	5.528
MAE (Str 5)	**4.920**	4.925	5.002

**Table 11:** MAEs of different facial age estimation algorithms.

Method	MAE
**SDM-LDL(Str 1)**	5.137
**SDM-LDL(Str 2)**	5.127
**SDM-LDL(Str 3)**	5.112
**SDM-LDL(Str 4)**	5.089
**SDM-LDL(Str 5)**	4.920
Original LDL	5.32
Hierarchical Framework [29]	4.97
LBP Kernel Density Estimate [30]	5.09
Local radon Features [31]	6.18
Cumulative Attribute SVR [32]	4.67
Grassmann Manifold [33]	5.89
Hierarchical Model [34]	4.89
Ordinal Hyperplanes Ranker (OHRank) [18]	6.27
Shape-based age estimation [35]	6.2
Regression using a learned distance metric [36]	5.04
Bio-inspired Features [26]	4.77
Synchronized Submanifold Embedding [37]	5.21
Manifold Learning and Locally Adjusted Robust Regressor [5]	5.07
Facial Aging Patterns (AGES) [10]	6.77
SVM	7.25
kNN	8.24

## References

[1] How-old.net Microsoft. http://how-old.net.

[2] Electronic Customer Relationship Management (ECRM). http://en.wikipedia.org/wiki/ECRM.

[3] Kloeppel J.E. Step Right up, Let the Computer Look at Your Face and Tell You Your Age. http://news.illinois.edu/news/08/0923age.html.

[4] Dix A., Finlay J., Abowd G.D., Beale R. Human-Computer Interaction. http://fit.mta.edu.vn/files/DanhSach/__Human_computer_interaction.pdf.

[5] Guo G., Fu Y., Dyer C., Huang T. (2008). Image-based human age estimation by manifold learning and locally adjusted robust regression. IEEE Trans. Image Process..

[6] Ramanathan N., Chellappa R. (2006). Face verification across age progression. IEEE Trans. Image Process..

[7] Albert A.M., Ricanek K., Pattersonb E. (2007). A review of the literature on the aging adult skull and face: Implications for forensic science research and applications. Forensic Sci. Int..

[8] Weng R., Lu J., Yang G., Tan Y. Multi-feature ordinal ranking for facial age estimation. Proceedings of the IEEE International Conference and Workshops on Automatic Face and Gesture Recognition.

[9] Wang C., Su Y., Hsu C., Lin C., Liao H. Bayesian age estimation on face images. Proceedings of the IEEE Conf. on Multimedia and Expo.

[10] Geng X., Zhou Z., Smith-Miles K. (2007). Automatic age estimation based on facial aging patterns. IEEE Trans. Pattern Anal. Mach. Intell..

[11] Lanitis A., Draganova C., Christodoulou C. (2004). Comparing different classifiers for automatic age estimation. IEEE Trans. Syst. Man Cybernet. Part B.

[12] Yang Z., Ai H. Demographic classification with local binary patterns. Proceedings of the International Conference on Biometrics.

[13] Fu Y., Huang T. (2008). Human age estimation with regression on discriminative aging manifold. IEEE Trans. Multimedia.

[14] Zhang Y., Yeung D. Multi-task warped Gaussian process for personalized age estimation. Proceedings of the IEEE Conference on Computer Vision and Pattern Recognition.

[15] Ni B., Song Z., Yan S. Web image mining towards universal age estimator. Proceedings of the ACM International Conference on Multimedia.

[16] Xiao B., Yang X., Zha H., Xu Y., Huang T. (2009). Metric Learning for Regression Problems and Human Age Estimation. Advances in Multimedia Information Processing—PCM 2009.

[17] Guo G., Fu Y., Huang T., Dyer C. Locally adjusted robust regression for human age estimation. Proceedings of the IEEE Workshop on Applications of Computer Vision.

[18] Chang K., Chen C., Hung Y. Ordinal hyperplanes ranker with cost sensitivities for age estimation. Proceedings of the IEEE Conference on Computer Vision and Pattern Recognition.

[19] Yan S., Wang H., Huang T., Yang Q. Ranking with uncertain labels. Proceedings of the IEEE Conference on Multimedia and Expo.

[20] Chao W.L., Liu J.Z., Ding J.J. (2013). Facial age estimation based on label-sensitive learning and age-oriented regression. Pattern Recognit..

[21] Geng X., Yin C., Zhou Z.H. (2013). Facial age estimation by learning from label distributions. IEEE Trans. Pattern Anal. Mach. Intell..

[22] Geng X., Ji R. Label distribution learning. Proceedings of the 13th International Conference on Data Mining Workshops (ICDMW).

[23] The FG-NET Aging Database. http://www.fgnet.rsunit.com/.

[24] Berger A.L., Pietra S.D., Pietra V.J.D. (1996). A maximum entropy approach to natural language processing. Comput. Linguist..

[25] Cootes T.F., Edwards G.J., Taylor C.J. (2001). Active appearance models. IEEE Trans. Pattern Anal. Mach. Intell..

[26] Guo G., Mu G., Fu Y., Huang T. Human age estimation using bio-inspired features. Proceedings of the IEEE Conference on Computer Vision and Pattern Recognition.

[27] Yang P., Zhong L., Metaxas D. Ranking model for facial age estimation. Proceedings of the International Conference on Pattern Recognition.

[28] Yan S., Wang H., Tang X., Huang T. Learning auto-structured regressor from uncertain nonnegative labels. Proceedings of the International Conference on Computer Vision.

[29] Liang Y., Wang X., Zhang L., Wang Z. (2014). A hierarchical framework for facial age estimation. Math. Probl. Eng..

[30] Ylioinas J., Hadid A., Hong X., Pietikainen M. Age estimation using local binary pattern kernel density estimate. Proceedings of the 17th International Conferenceon Image Analysis and Processing.

[31] Gunay A., Nabiyev V.V. (2013). Age estimation based on local radon features of facial images. Computer and Information Sciences III.

[32] Chen K., Gong S., Xiang T., Loy C.C. Cumulative attribute space for age and crowd density estimation. Proceedings of the IEEE Conference on Computer Vision and Pattern Recognition.

[33] Wu T., Turaga P., Chellappa R. (2012). Age estimation and face verification across aging using landmarks. IEEE Trans. Inf. Forensics Secur..

[34] Zhang L., Wang X., Liang Y., Xie L. A new method for age estimation from facial images by hierarchical model. Proceedings of the International Conference on Innovative Computing and Cloud Computing.

[35] Thukral P., Mitra K., Chellappa R. A hierarchical approach for human age estimation. Proceedings of the IEEE Internationl Conference on Acoustics, Speech and Signal Processing.

[36] Xiao B., Yang X., Xu Y., Zha H. Learning distance metric for regression by semidefinite programming with application to human age estimation. Proceedings of the ACM Internationl Conference on Multimedia.

[37] Yan S., Wang H., Fu Y., Yan J., Tang X., Huang T.S. (2009). Synchronized submanifold embedding for person-independent pose estimation and beyond. IEEE Trans. Image Process..

